# Newborn body composition and child cardiovascular risk markers: a prospective multi-ethnic Asian cohort study

**DOI:** 10.1093/ije/dyac154

**Published:** 2022-07-30

**Authors:** Yi Ying Ong, Mya-Thway Tint, Izzuddin M. Aris, Wen Lun Yuan, Ling-Wei Chen, Marielle V. Fortier, Jonathan Choo, Lieng Hsi Ling, Lynette Shek, Kok Hian Tan, Peter D. Gluckman, Fabian Yap, Yap-Seng Chong, Keith M. Godfrey, Mary F-F. Chong, Shiao-Yng Chan, Johan G. Eriksson, Mary E. Wlodek, Emanuella De Lucia Rolfe, Ken K. Ong, Navin Michael, Yung Seng Lee

**Affiliations:** 1 Department of Paediatrics, Yong Loo Lin School of Medicine, National University of Singapore, Singapore, Singapore; 2 Department of Obstetrics and Gynaecology and Human Potential Translational Research Programme, Yong Loo Lin School of Medicine, National University of Singapore, Singapore, Singapore; 3 Singapore Institute for Clinical Science, Agency for Science, Technology, and Research, Singapore, Singapore; 4 Institute of Epidemiology and Preventive Medicine, College of Public Health, National Taiwan University, 17 Hsu-Chow Road, Taipei 100, Taiwan; 4 Division of Chronic Disease Research Across the Lifecourse, Department of Population Medicine, Harvard Medical School and Harvard Pilgrim Health Care Institute, Boston, Massachusetts, USA; 5 Department of Diagnostic and Interventional Imaging, KK Women’s and Children’s Hospital, Singapore, Singapore; 6 Department of Paediatrics, KK Women’s and Children’s Hospital, Singapore, Singapore; 7 Department of Cardiology, National University Heart Centre, Singapore, Singapore; 8 Division of Paediatric Endocrinology, Department of Paediatrics, Khoo Teck Puat-National University Children’s Medical Institute, National University Hospital, National University Health System, Singapore; 9 Duke-NUS Medical School, Singapore, Singapore; 10 Department of Maternal Fetal Medicine, KK Women’s and Children’s Hospital, Singapore, Singapore; 11 Liggins Institute, University of Auckland, Auckland, New Zealand; 12 MRC Lifecourse Epidemiology Unit and NIHR Southampton Biomedical Research Centre, University of Southampton and University Hospital Southampton NHS Foundation Trust, Southampton, UK; 13 Saw Swee Hock School of Public Health, National University of Singapore, Singapore, Singapore; 14 Department of General Practice and Primary Health Care, University of Helsinki, Helsinki, Finland; 15 Folkhälsan Research Center, Helsinki, Finland; 17 Medical Research Council Epidemiology Unit, Institute of Metabolic Science, University of Cambridge, Cambridge, United Kingdom

**Keywords:** Adiposity, Fetal growth, Birthweight, Blood pressure, Cardiovascular

## Abstract

**Background:**

Early epidemiological studies have associated low birthweight with increased cardiovascular risk. We aimed to examine whether the fat and fat-free components of birthweight have differing relationships with childhood cardiovascular risk markers.

**Methods:**

In the Growing Up in Singapore Towards healthy Outcomes (GUSTO) cohort, air displacement plethysmography was conducted within 24 hours after delivery in 290 naturally-conceived singletons. We investigated associations of newborn cohort-specific standardized z-score of fat mass, fat-free mass, body fat percentage, and birthweight on child (6y) carotid intima-media thickness, pulse wave velocity, blood pressure, prehypertension/hypertension (>110/70mmHg), and standardized systolic and diastolic blood pressure (SBP and DBP) trajectories (3-6y), taking account of maternal education, height, tobacco exposure, parity, ethnicity, child’s sex, gestational age, age at follow-up, and other maternal factors.

**Results:**

Clear inverse associations were seen for blood pressure with z-score of fat mass [SBP, β (95% CI): -1.31mmHg (-2.57, -0.06); DBP: -0.79mmHg (-1.74, 0.15)] and body fat percentage [SBP: - 1.46mmHg (-2.73, -0.19); DBP: -0.80mmHg (-1.75, 0.16)], but not with fat-free mass [SBP: 0.27mmHg (-1.29, 1.83)]; DBP: -0.14mmHg (-1.30, 1.03)]. Being in the lowest tertile of fat mass or body fat percentage was associated with higher blood pressure trajectories and prehypertension/hypertension risk [OR (95% CI), fat mass: 4.23 (1.41, 12.68); body fat percentage: 3.22 (1.09, 9.53)] without concomitantly higher overweight/obesity risk.

**Conclusions:**

At birth, low adiposity was associated with increased childhood blood pressure. Low newborn adiposity might serve as a marker of poor fetal growth or suboptimal intrauterine conditions associated with hypertension risk later in life.

## Introduction

Cardiovascular diseases are becoming increasingly prevalent worldwide, representing a major public health problem. Evidence from early epidemiologic studies have associated poor fetal growth/nutrition with greater cardiovascular risk later in life ^1,2^, which might be explained by various mechanisms such as reduced nephron numbers, reduced elastin reserves (a key protein enabling blood vessel elasticity), and altered programming of the renin-angiotensin system ^3^. Birthweight or ponderal index are commonly used as proxies of poor fetal growth/nutrition and have been associated with cardiovascular risk later in life ^4,5^.

However, several gaps exist in this field. Birthweight alone might not be the best proxy of fetal growth/nutrition because it is influenced by genetic factors and might not be able to differentiate constitutionally small infants from those who are truly growth restricted ^6^. According to mendelian randomization analyses, genetically determined low birthweight was not associated with blood pressure, but this does not exclude the possibility of environmentally/nutritionally determined low birthweight being associated with cardiovascular risk ^7^. Furthermore, earlier studies which used birthweight or ponderal index as crude proxies of poor fetal growth/nutrition were unable to differentiate between fat mass and lean mass, which represent two physiologically distinct components of the body ^8,9^and thus might have differing associations with cardiovascular risk. For instance, reduced fat-free mass might track to adulthood and contribute to increased cardiovascular risk due to the key role of lean mass in regulating energy expenditure and progression of cardiometabolic diseases ^9^. In contrast, increased fat mass at birth is thought to be detrimental to cardiometabolic health as neonatal adiposity might track to childhood and adulthood ^10,11^. However, decreased relative fat mass might be a sensitive marker of fetal undernutrition ^12–15^, and fetal undernutrition is associated with increased cardiovascular risk, especially if it is coupled with rapid postnatal weight gain ^16^. Therefore, it is unclear how whole-body fat mass, fat-free mass, and body fat percentage at birth are differentially associated with cardiovascular risk. To the best of our knowledge, no previous studies have investigated associations between newborn body composition measured by air displacement plethysmography and childhood cardiovascular risk markers.

To address this important gap in cardiovascular and public health research, we aim to investigate the associations between newborn body composition and cardiovascular risk in a prospective multi-ethnic cohort, using precise measurements from air displacement plethysmography which has a good agreement with the 4-compartment gold standard model of assessing body composition in humans ^17^. Following up these children till 6 years old, we investigated an extensive panel of child cardiovascular risk markers (blood pressure trajectories, carotid intimamedia thickness, pulse wave velocity, prehypertension/hypertension risk, etc.) to capture early changes in various biomarkers related to cardiovascular profile during early childhood. We hypothesize that newborn fat mass and fat-free mass are differentially associated with cardiovascular risk at 6 years old, hence providing additional insights for child cardiovascular risk stratification compared to birthweight alone.

## Materials and methods

### Study population

From June 2009 to October 2010, pregnant women in their first trimester were recruited from KK Women’s and Children’s Hospital (KKH) and National University Hospital (NUH). Eligibility criteria include Singapore citizens/permanent residents aged at least 18 years, of homogenous parental ethnic background, planned to deliver in KKH/NUH and reside in Singapore for the next 5 years, and willing to donate birth tissues at delivery. Women receiving chemotherapy, on psychotropic drugs, or having type 1 diabetes were excluded. Of 1097 naturally conceived singletons delivered, a subset of 318 infants whose parents consented and who were born in KKH underwent air displacement plethysmography at birth. Our study included 290 of these infants who were followed-up till age 6 years (Figure S1). Approval from the National Healthcare Group Domain Specific Review Board and SingHealth Centralized Institutional Review Board and written informed consent from participants were obtained.

### Exposure

Birthweight was obtained from medical records. Within 24 hours after delivery, newborn body composition was measured by air displacement plethysmography (PEAPOD Infant Body Composition System Version 3.1.0 Cosmed, Rome, Italy). Daily calibration of the system was carried out. Before measurement, clothing was removed and a tight-fitting cap was worn. Total body volume was measured by the PEAPOD chamber, adjusted for thoracic gas volume and surface area artifact ^18^. Using a fat density of 0.9007 kg/L and an age and sex-specific fat-free density ^19^, the PEAPOD system calculates the fat mass, fat free mass, and body fat percentage. To ensure comparability between the different exposure measures, cohort-specific standardized z-scores of birthweight (z-birthweight), fat-free mass (z-fat free mass), fat mass (z-fat mass), and body fat percentage (z-body fat percentage) were calculated.

### Child Outcomes

At age 6 years, child height (SECA 213 stadiometer) and weight (SECA 803 Weighing Scale) were measured to calculate sex- and age-standardized z-scores for weight, height (z-height), and body mass index (z-BMI) using World Health Organization growth standards ^20^. Overweight and obesity were defined as having a BMI of 1 and 2 standard deviations above the World Health Organization growth standard median respectively ^21^. Annually from ages 3 to 6 years, peripheral systolic blood pressure (SBP) and diastolic blood pressure (DBP) were measured from the right upper arm (Dinamap CARESCAPE V100, GE Healthcare, Milwaukee, WI) by trained research staff in a quiet room using standardized protocols. SBP and DBP were measured in duplicate and averaged. Children were identified as having “prehypertension/hypertension” if their blood pressures crossed the simplified pediatric threshold of 110/70 mmHg ^22^. Carotid intima-media thickness (cIMT) was measured using high resolution B-mode ultrasound (CX-50 XMatrix, Philips Medical Ultrasound Systems at KKH and Aloka at NUH) at the right common carotid artery 1 cm proximal to the carotid bulb. With the child in the supine position, applanation tonometry (SphygmoCorVx, AtCor Medical, West Ryde, NSW, Australia) was conducted to derive carotid-femoral pulse wave velocity (PWV) from the carotid-femoral path length and carotid-femoral transit time.

### Covariates

At recruitment, maternal age, ethnicity, highest educational attainment, household income, and self-reported pre-pregnancy weight were collected through interviewer-administered questionnaires. At the 26-28 week antenatal visit, maternal height (SECA213 Stadiometer, SECA Corp, Hamburg, Germany) and venous fasting plasma glucose [Advia 2400 Chemistry system (Siemens Medical Solutions Diagnostics, Deerfield, IL, USA) and Beckman LX20 Pro analyser (Beckman Coulter, USA)] were measured. Tobacco exposure was assessed through plasma cotinine and interviewer-administered questionnaires ^23^. Total gestational weight gain was calculated from pre-pregnancy weight and last measured antenatal weight. Gestational age was calculated from the first trimester ultrasound scan. Infant sex and maternal hypertensive disorders (including chronic hypertension, pregnancy-induced hypertension, and pre-eclampsia) were obtained from medical records. In a subset of neonates (N=160) whose parents consented, neonatal abdominal adiposity, namely the subcutaneous adipose tissue, superficial subcutaneous adipose tissue, deep subcutaneous adipose tissue, and intraabdominal adipose tissue volumes were measured by magnetic resonance imaging using a GE Signa HDxt 1.5T magnetic resonance scanner (GE Healthcare) within 2 weeks from delivery.

### Statistical analysis

All analyses were performed using Stata16.0 (StataCorp LP, TX). To compare differences between included and excluded participants, two-tailed t-tests and chi-square tests were performed. The linearity assumption was satisfied after testing for non-linearity by including spline terms ^24^. Multiple linear regression models were used to investigate the association between newborn body composition markers (birthweight, fat-free mass, fat mass, body fat %) and child cardiovascular risk markers (z-BMI, z-height, SBP, DBP, cIMT, PWV), adjusted for confounders (sex, ethnicity, maternal education, parity, maternal height, tobacco exposure, gestational age, exact age of child at year 6 visit, pre-pregnancy BMI, gestational fasting plasma glucose, gestational weight gain, maternal hypertension) to understand associations independent of these factors which are well-known to be associated with birthweight and cardiovascular risk ^25–27^. Since blood pressure in growing children is influenced by their sex and height ^26^, standardized residuals of blood pressure regressed on sex and height from 3-6 years old were calculated. Longitudinal associations between newborn body composition tertiles and standardized residuals of blood pressure were evaluated by linear mixed effects modeling using maximum likelihood estimation, assuming outcome data was missing at random ^28^, with unstructured covariances ^29^. Models included a random intercept, random linear slope for age, an age-body composition tertile interaction term, and the same confounders as the regression model above. Linear mixed effects-predicted standardized blood pressure for the body composition tertiles, while holding covariates constant at their mean values, were visualized. Logistic regression models were used to evaluate the odds of prehypertension/hypertension and overweight/obesity associated with being in the lowest tertile (tertile 1) or highest tertile (tertile 3) of newborn body composition compared to the middle tertile (tertile 2).

In sensitivity analyses, preterm infants (n=9, Gestational age: 35-36.86 weeks) were excluded. Secondly, to account for potential selection bias due to loss to follow-up and exclusion of participants who lack newborn body composition measurement, we fitted weighted linear regression models with inverse probability weighting using stabilized inverse probability weights ^30^, adjusted for the same confounders as above. Stabilized inverse probability weights were derived using the formula adapted from Hernán and Robins ^31^:

$$\;Stabilized\;inverse\;probability\;weights\;=\frac{Pr(A=1)}{Pr(A=1|L)}$$

Where Pr(A=1) stands for the marginal probability of being included and Pr(A=1|L) stands for the probability of being included conditional on a list of covariates, L, that included all confounders and baseline characteristics, with both the linear as well as quadratic term for all continuous variables. These probabilities were calculated by fitting logistic regression models for 791 participants who had all baseline confounders measured. We truncated the stabilized inverse probability weights at the 1^st^ and 99^th^ percentiles to exclude extreme weights.

## Results

### Cohort description

Of 318 infants who had body composition measured by PEAPOD, 290 were followed up at age 6 years (follow-up rate: 91.2%) and were included in this study (Figure S1). The study consisted of 46.2% Chinese, 36.6% Malay, and 17.2% Indian participants, with a mean ± standard deviation maternal age of 30.4 ± 5.5 years old (Table 1). Newborns had a mean gestational age of 38.9 ± 1.1 weeks and birthweight of 3.12 ± 0.38 kg. Compared to included participants, excluded participants had higher maternal age (31.1 ± 5.0y), were more likely to be Chinese, had higher maternal education, higher household income, higher gestational 2-hour plasma glucose (6.57 ± 1.46mmol/L), were more likely to have mothers with gestational diabetes, had lower tobacco exposure, and lower gestational age (38.7 ± 1.7wk) (Table S1). Neonates in the lowest tertiles of body composition markers (birthweight, fat-free mass, fat mass, or body fat percentage) also had the lowest neonatal abdominal adiposity across all compartments measured compared to those in the middle and highest tertiles (Table S2).

### Newborn body composition and cardiovascular risk markers

**Figure 1:**
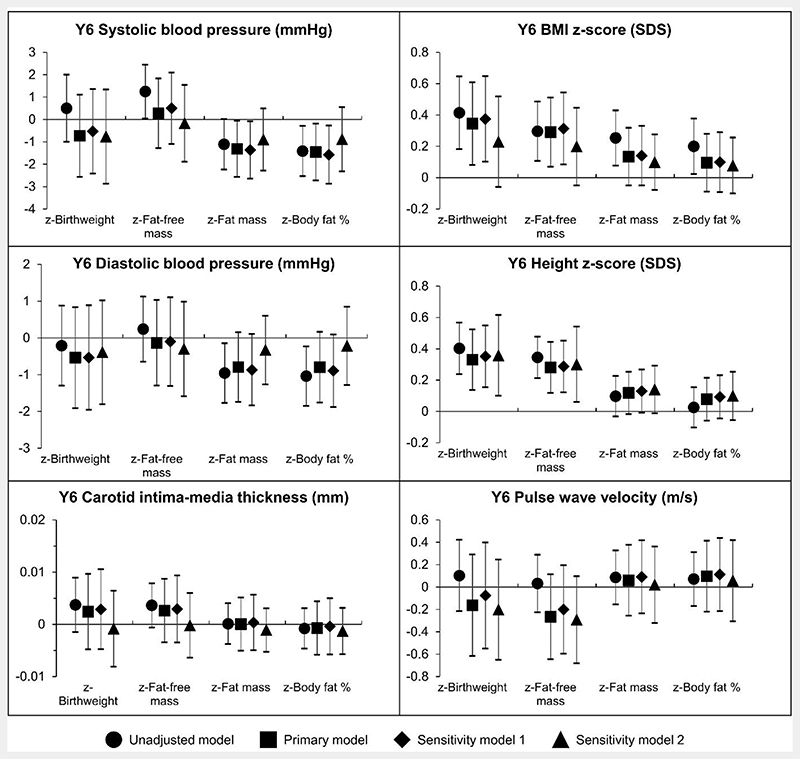
Association between z-scores of newborn body composition markers and cardiovascular risk markers at 6 years old. Regression coefficients with 95% confidence intervals are shown. The primary model is adjusted for potential confounders (sex, ethnicity, maternal education, parity, maternal height, tobacco exposure, gestational age, age at year 6 visit, pre-pregnancy body mass index, gestational diabetes, gestational weight gain, and maternal hypertension). Adjusting for the same list of confounders, sensitivity model 1 excludes preterm infants, while sensitivity model 2 show inverse probability weighted estimates. Legend: SDS = standard deviation score

**Figure 2:**
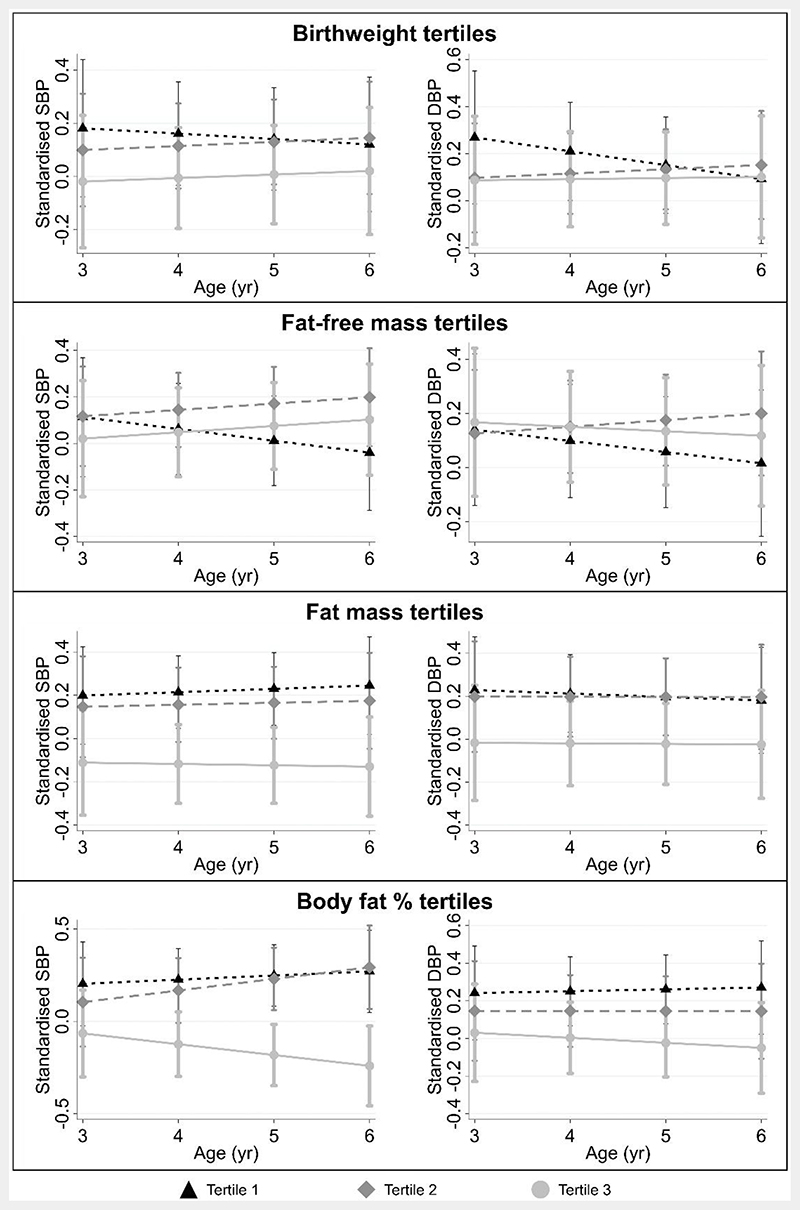
Standardized blood pressure between ages 3-6 years, across tertiles of newborn body composition markers. Linear mixed models were adjusted for sex, ethnicity, maternal education, parity, maternal height, tobacco exposure, gestational age, pre-pregnancy body mass index, gestational diabetes, gestational weight gain, and maternal hypertension. Predicted standardized blood pressures (error bars showing 95% CI) while holding all other covariates at their mean values are shown. Legend: SBP = systolic blood pressure; DBP = diastolic blood pressure

Adjusting for confounders, newborn z-fat mass and z-body fat percentage were inversely associated with SBP [z-fat mass: -1.31 mmHg (-2.57, -0.06); z-body fat percentage: -1.46 mmHg (-2.73, -0.19)] and DBP [z-fat mass: -0.79 mmHg (-1.74, 0.15); z-body fat percentage: -0.80 mmHg (-1.75, 0.16)] at 6 years of age (Figure 1). Being in the highest tertile of newborn fat mass or body fat percentage was consistently associated with having the lowest blood pressure trajectories (Figure 2) while being in the lowest tertile of newborn fat mass or body fat percentage was associated with increased odds of prehypertension/hypertension at 6 years old [OR (95% CI), fat mass: 4.23 (1.41, 12.68); body fat percentage: 3.22 (1.09, 9.53)] (Table 2). Despite newborn adiposity being associated with blood pressure, no clear associations were observed with cIMT, PWV, z-BMI, z-height, or overweight/obesity risk.

In contrast, z-fat-free mass was positively associated with z-BMI [0.29 SDS (0.07, 0.51)] and z-height [0.28 SDS (0.12, 0.44)] but not clearly associated with SBP [0.27 mmHg (-1.29, 1.83)] and DBP [-0.14 mmHg (-1.30, 1.03)] at 6 years of age.

### Sensitivity analyses

After excluding preterm infants, associations remained similar (Figure 1). After inverse probability weighting, associations remained similar, but the magnitude of associations between newborn adiposity and blood pressure were decreased.

## Discussion

From our prospective mother-offspring cohort, we examined the fat and fat-free components of birthweight which might provide additional insights on how low birthweight, a proxy of poor fetal growth, is associated with increased cardiovascular risk. Indeed, newborn fat mass and fat-free mass had differing associations with cardiovascular risk markers, whereby newborn fat mass, but not fat-free mass, was inversely associated with blood pressure at age 6 years. In contrast, no clear associations were observed between birthweight and blood pressure. Our findings suggest the value of examining newborn body composition rather than birthweight alone for early cardiovascular risk stratification. Associations between low newborn body fat percentage and increased cardiovascular risk markers suggest that disproportionately lower fetal fat mass relative to fat-free mass might be a marker for specific intrauterine conditions that program the risk of hypertension later in life. Notably, associations between low newborn adiposity and increased child blood pressure were observed without concomitantly increased child overweight/obesity and without increased arterial thickness/stiffness. Perhaps alternative pathways such as reduction in nephron number or altered programming of the renin-angiotensin system may be involved in explaining the association between low newborn adiposity and increased child blood pressure.

We did not find a clear relationship between birthweight and cardiovascular risk markers at age 6 years. Firstly, this could be because in our cohort, most children were not severely growth restricted in the extremes of birthweight as participants resided in a modern, well-developed country with no drastic nutritional or environmental stresses, which could have led to a smaller effect size. Secondly, while inverse associations between birthweight and blood pressure have been reported in adults ^32^, findings in children have been mixed ^33^, perhaps because effects of low birthweight on cardiovascular risk would only be amplified over time with greater weight gain and metabolic load experienced over the course of life ^34^. Thirdly, previous studies which observed associations between birthweight and blood pressure after adjustment for child’s current body size might be affected by the reversal paradox, which arises from inappropriate adjustment for variables, such as current body size, which might lie on the causal pathway between birthweight and blood pressure ^35^.

In contrast, low newborn adiposity was clearly associated with increased cardiovascular risk. Association between low newborn adiposity and prehypertension/hypertension risk might be due to lower fetal fat accumulation ^36^ and lower adiposity at birth ^17,37^ being reflective of poor fetal growth/nutrition or genetic predisposition to lower quantity of metabolically favorable adiposity ^38^, which might be associated with increased hypertension risk. Previous studies have suggested that associations between low neonatal adiposity and higher cardiovascular risk markers might also be driven by decreased deposition of the more metabolically favourable subcutaneous adipose tissue mass ^39^. However, the role of metabolically favourable fat (e.g., subcutaneous fat) or metabolically unfavourable fat (e.g., intraabdominal fat) in driving cardiovascular risk is not clearly seen in our study. We found that neonates in the lowest tertile of overall fat mass had increased prehypertension/hypertension risk, the least subcutaneous abdominal fat, but also the least intraabdominal fat compared to those in the middle or highest tertiles. Additionally, while neonates in the lowest tertile of fat-free mass also had the least subcutaneous abdominal fat compared to those in the middle or highest tertiles, they did not have increased prehypertension/hypertension risk. Further investigations to elucidate the pathways between low neonatal adiposity and increased cardiovascular risk are needed. The inverse relationship between newborn adiposity and child blood pressure was observed to exist linearly across the continuum, where higher newborn adiposity was associated with lower blood pressure. This might seem contrary to the traditional notion that high newborn adiposity is not favorable as it might be associated with both higher childhood adiposity ^10,11^ as well as higher blood pressure/cardiovascular risk ^40^. The conflicting findings between adiposity and blood pressure might be due to the contrasting roles of newborn adiposity vs. childhood adiposity. While high childhood adiposity might act as a marker of postnatal overnutrition and contribute to the metabolic load of the body ^41^, thus contributing to increased cardiometabolic risk, low newborn adiposity might be a marker of poor fetal growth/nutrition in late gestation ^12^, potentially explaining its association with increased blood pressure in childhood. Furthermore, lower neonatal body fat percentage has been suggested to be a more sensitive marker of fetal undernutrition than small-for-gestational age due to its stronger correlation with neonatal morbidity ^14,15^. Since it is established that poor fetal growth is associated with increased cardiovascular risk through various mechanisms, low newborn body fat percentage might be an indicator of poor fetal growth, explaining its strong association with blood pressure and cardiovascular risk.

Previous studies have suggested that increased arterial thickness, stiffness ^42^, and obesity ^27^ might explain the association between poor fetal growth and increased blood pressure in adulthood. Small-for-gestational-age and intrauterine growth restricted children were found to have increased blood pressure and cIMT at 3-6 years old ^43^. Lower birthweight has also been associated with increased PWV ^44^. However, in our study, the association of low newborn adiposity with increased blood pressure at 6 years old was not explained by concurrent child obesity or physical changes to the arteries. The contrasting findings with previous studies might be due to different exposures investigated, since we studied low body fat percentage, unlike previous studies which studied low birthweight, small-for-gestational-age, or intrauterine growth restriction. Hence, there might be a different etiology of elevated blood pressure. The lack of association with childhood cIMT and PWV at 6 years old might also be because these markers may primarily reflect normal growth-related influences rather than pathophysiology at this stage of life. Furthermore, there might be greater measurement error or small variability in these measures in young children ^45,46^, and associations might emerge later in childhood ^40^.

Our study has several strengths and limitations. To the best of our knowledge, our study was the first to investigate the fat and fat-free mass components of birthweight and newborn body fat percentage, measured precisely using air displacement plethysmography, on cardiovascular markers such as blood pressure trajectories, cIMT and PWV. Other strengths include our prospective study design, extensive adjustment for confounders, sensitivity analyses to ensure the robustness of our findings, and inverse probability weighting to reduce selection bias. Limitations include the possibility of bias from residual confounding and possibility of measurement error which might partly explain the lack of associations in measures with small variability. While multiple related cardiovascular outcomes were selected *a priori* based on literature ^2,47^ and with plausible biological mechanisms ^3,48^, which reduces the risk of false positives arising simply by chance, we acknowledge that the lack of multiple testing correction for the multiple related cardiovascular outcomes might increase the risk of false positives. The change in effect estimates after inverse probability weighing suggest that findings in the primary model might only apply to this selected sample or to populations with similar baseline characteristics presented in Table 1 but might not be generalizable to the entire GUSTO cohort due to selection bias. Finally, our study was conducted in a multi-ethnic Asian cohort among naturally-conceived singletons, so findings might not be generalizable to other populations.

In conclusion, while early epidemiological studies have associated low birthweight or low ponderal index with increased cardiovascular risk, our study sheds insight on the differing relationships of newborn fat and fat-free mass on blood pressure and highlights the potential of low newborn adiposity as a proxy of poor fetal growth/nutrition which might provide additional value for cardiovascular risk stratification compared to birthweight alone. There is a paucity of studies on newborn body composition and our study provides impetus for further investigations on newborn adiposity and fat partitioning due to their potential associations with altered cardiovascular risk markers manifesting even in young children, which might eventually contribute to the rising incidence of cardiovascular diseases worldwide. Further studies to understand if pathways such as low nephron number, hormonal, genetic, or epigenetic pathways explain these associations would be valuable.

## Supplementary Material

S1.

## Figures and Tables

**Table 1:** Demographic and clinical characteristics of participants.

	Mean ± SD / N (%)
**Parental characteristics**	
Maternal age (yr)	30.4 ± 5.5
Ethnicity	
Chinese	134 (46.2%)
Malay	106 (36.6%)
Indian	50 (17.2%)
Maternal education	
University	66 (23.0%)
Post-secondary	104 (36.2%)
Secondary or lower	117 (40.8%)
Household income (monthly)	
Low (< $4000)	152 (56.5%)
Mid ($4000-$5999)	69 (25.7%)
High (≥ $6000)	48 (17.8%)
Pre-pregnancy BMI (kg/m^2^)	23.2 ± 5.0
Maternal height (cm)	158 ± 6
Total gestational weight gain	
Normal	81 (33.3%)
Inadequate	70 (28.8%)
Excessive	92 (37.9%)
Gestational fasting plasma glucose (mmol/L)	4.38 ± 0.40
Gestational 2-hour plasma glucose (mmol/L)	6.30 ± 1.42
Gestational diabetes	
No	244 (87.5%)
Yes	35 (12.5%)
Maternal hypertension	
None	269 (92.8%)
Pregnancy-induced hypertension	9 (3.1%)
Pre-eclampsia	7 (2.4%)
Chronic hypertension	5 (1.7%)
Parity	
Parous	175 (60.3%)
Nulliparous	115 (39.7%)
Tobacco exposure groups^a^	
Group 1	120 (43.0%)
Group 2	93 (33.3%)
Group 3	53 (19.0%)
Group 4	13 (4.7%)
**Neonatal characteristics**	
Gestational age (wk)	38.9 ± 1.1
Sex	
Female	143 (49.3%)
Male	147 (50.7%)
Birthweight (kg)	3.12 ± 0.38
Fat-free mass (kg)	2.77 ± 0.31
Fat mass (kg)	0.31 ± 0.14
Body fat %	9.91 ± 3.58
Girls	10.68 ± 3.79
Boys	9.15 ± 3.21
**6-year-old child characteristics**	
z-BMI (SDS)	0.17 ± 1.40
z-Height (SDS)	-0.17 ± 1.02
Systolic blood pressure (mmHg)	102 ± 9
Diastolic blood pressure (mmHg)	60 ± 6
Carotid intima media thickness (mm)	0.42 ± 0.03
Pulse wave velocity (m/s)	5.07 ± 1.62

Legend: SD = standard deviation; BMI = body mass index; SDS = standard deviation score

a Group 1: cotinine <0.17 ng/mL and no environmental tobacco smoke exposure; Group 2: cotinine <0.17 ng/mL but self-reported environmental tobacco smoke exposure; Group 3: cotinine 0.17–13.99 ng/mL (environmental tobacco smoke exposure or light smoking); Group 4: cotinine ≥14 ng/mL (active smoking)

**Table 2:** Odds of prehypertension/hypertension (> 110/70 mmHg) and overweight/obesity (z-BMI > 1) at age 6 years associated with being in the lowest tertile (tertile 1) or highest tertile (tertile 3) of newborn body composition markers (birthweight, fat mass, fat-free mass, body fat %), with reference to the middle tertile (tertile 2).

	Unadjusted OR (95% CI)	Adjusted^a^ OR (95% CI)	Adjusted^b^ OR (95% CI)	Adjusted^c^ OR (95% CI)
**Odds of Y6 prehypertension or hypertension**				
Birthweight				
Tertile 1	0.54 (0.22, 1.35)	0.75 (0.23, 2.44)	0.75 (0.23, 2.46)	0.86 (0.19, 3.89)
Tertile 2	ref.	ref.	ref.	ref.
Tertile 3	0.67 (0.30, 1.48)	0.54 (0.18, 1.63)	0.54 (0.18, 1.65)	0.54 (0.12, 2.34)
Fat-free mass				
Tertile 1	0.32 (0.11, 0.92)	0.39 (0.11, 1.43)	0.40 (0.11, 1.46)	0.81 (0.17, 3.95)
Tertile 2	ref.	ref.	ref.	ref.
Tertile 3	1.07 (0.50, 2.29)	1.20 (0.44, 3.26)	1.21 (0.44, 3.30)	1.51 (0.38, 5.92)
Fat mass				
Tertile 1	2.37 (1.01, 5.55)	4.23 (1.41, 12.68)	4.21 (1.40, 12.64)	3.13 (0.90, 10.91)
Tertile 2	ref.	ref.	ref.	ref.
Tertile 3	0.91 (0.35, 2.34)	0.67 (0.18, 2.44)	0.66 (0.18, 2.42)	0.52 (0.11, 2.60)
Body fat %				
Tertile 1	1.80 (0.80, 4.04)	3.22 (1.09, 9.53)	3.21 (1.08, 9.51)	2.49 (0.62, 9.92)
Tertile 2	ref.	ref.	ref.	ref.
Tertile 3	0.45 (0.17, 1.22)	0.25 (0.06, 1.12)	0.25 (0.06, 1.10)	0.27 (0.04, 1.82)
**Odds of Y6 overweight or obesity**				
Birthweight				
Tertile 1	0.65 (0.29, 1.47)	0.52 (0.17, 1.54)	0.50 (0.16, 1.53)	0.42 (0.14, 1.27)
Tertile 2	ref.	ref.	ref.	ref.
Tertile 3	1.52 (0.78, 2.98)	1.29 (0.46, 3.61)	1.33 (0.48, 3.73)	1.35 (0.40, 4.51)
Fat-free mass				
Tertile 1	0.57 (0.26, 1.23)	0.28 (0.09, 0.86)	0.28 (0.09, 0.87)	0.24 (0.07, 0.82)
Tertile 2	ref.	ref.	ref.	ref.
Tertile 3	1.12 (0.57, 2.21)	1.02 (0.38, 2.74)	1.06 (0.40, 2.86)	0.94 (0.30, 2.99)
Fat mass				
Tertile 1	0.74 (0.35, 1.56)	0.62 (0.23, 1.69)	0.60 (0.22, 1.64)	0.58 (0.18, 1.88)
Tertile 2	ref.	ref.	ref.	ref.
Tertile 3	1.14 (0.56, 2.30)	0.65 (0.24, 1.75)	0.66 (0.25, 1.77)	0.69 (0.19, 2.50)
Body fat %				
Tertile 1	1.42 (0.65, 3.11)	1.64 (0.57, 4.70)	1.58 (0.55, 4.53)	1.69 (0.50, 5.73)
Tertile 2	ref.	ref.	ref.	ref.
Tertile 3	2.29 (1.07, 4.89)	1.57 (0.56, 4.39)	1.55 (0.56, 4.34)	1.98 (0.55, 7.10)

Legend: OR = odds ratio; CI = confidence interval

a Primary model: adjusted for sex, ethnicity, maternal education, parity, maternal height, tobacco exposure, gestational age, age at year 6 visit, pre-pregnancy body mass index, gestational diabetes, gestational weight gain, and maternal hypertension

b Sensitivity model 1: primary model excluding preterm infants

c Sensitivity model 2: primary model with inverse probability weighted estimates

## Data Availability

Restrictions apply to the availability of some or all data generated or analyzed during this study to preserve patient confidentiality or because they were used under license. The corresponding author will on request detail the restrictions and any conditions under which access to some data may be provided.

## Key messages

- Low newborn fat mass was associated with elevated blood pressure trajectories from 3-6 years old as well as increased blood pressure and prehypertension/hypertension risk at 6 years old.
- The clear inverse associations between newborn adiposity and blood pressure were not observed with newborn fat-free mass or birthweight.
- Assessing newborn body composition might provide additional value for cardiovascular risk stratification compared to birthweight alone.
- Low newborn adiposity might serve as a proxy of poor fetal growth/nutrition and a marker for specific intrauterine conditions that program higher blood pressure in 6-year-old children.
