# Supplementary material for: Newborn body composition and child cardiovascular risk markers: a prospective multi-ethnic Asian cohort study
Source: Int J Epidemiol. Author manuscript; Available in PMC 2022 Dec 15. (PMC9749728; doi:10.1093/ije/dyac154)
Supplement: S1. [file EMS152657-supplement-S1_.docx]

Figure S1: Participant recruitment flow chart and sample included in this study.


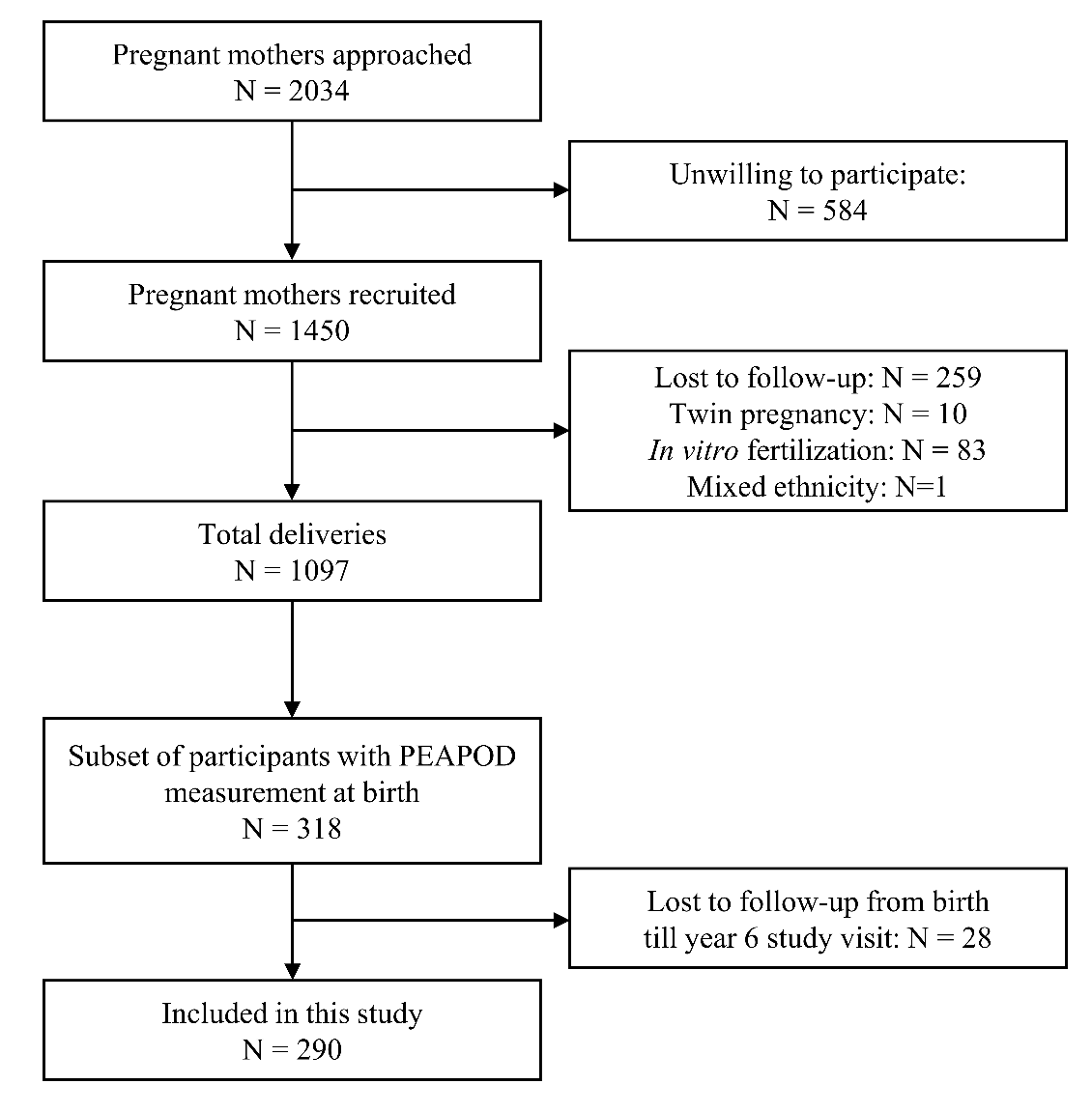


Table S1: Demographic and clinical characteristics of participants. Legend: BMI = body mass index; SDS = standard deviation score

|  | **Total**  **N=** **1097** | **Excluded**  **N=** **807** | **Included**  **N=290** | **p** |
| --- | --- | --- | --- | --- |
| **Parental characteristics** |  |  |  |  |
| Maternal age (yr) | 30.9 ± 5.1 | 31.1 ± 5.0 | 30.4 ± 5.5 | 0.033 |
| Ethnicity |  |  |  | <0.001 |
| Chinese | 599 (54.6%) | 465 (57.6%) | 134 (46.2%) |  |
| Malay | 292 (26.6%) | 186 (23.0%) | 106 (36.6%) |  |
| Indian | 206 (18.8%) | 156 (19.3%) | 50 (17.2%) |  |
| Maternal education |  |  |  | <0.001 |
| University | 356 (32.9%) | 290 (36.4%) | 66 (23.0%) |  |
| Post-secondary | 385 (35.5%) | 281 (35.3%) | 104 (36.2%) |  |
| Secondary or lower | 342 (31.6%) | 225 (28.3%) | 117 (40.8%) |  |
| Household income |  |  |  | <0.001 |
| Low (< $4000) | 487 (47.5%) | 335 (44.3%) | 152 (56.5%) |  |
| Mid ($4000-$5999) | 257 (25.0%) | 188 (24.8%) | 69 (25.7%) |  |
| High (≥ $6000) | 282 (27.5%) | 234 (30.9%) | 48 (17.8%) |  |
| Pre-pregnancy BMI (kg/m^2^) | 22.7 ± 4.4 | 22.6 ± 4.2 | 23.2 ± 5.0 | 0.052 |
| Maternal height (cm) | 158 ± 6 | 158 ± 6 | 158 ± 6 | 0.270 |
| Total gestational weight gain |  |  |  | 0.591 |
| Normal | 341 (36.0%) | 260 (37.0%) | 81 (33.3%) |  |
| Inadequate | 263 (27.8%) | 193 (27.5%) | 70 (28.8%) |  |
| Excessive | 342 (36.2%) | 250 (35.6%) | 92 (37.9%) |  |
| Gestational fasting plasma glucose (mmol/L) | 4.35 ± 0.47 | 4.34 ± 0.49 | 4.38 ± 0.40 | 0.215 |
| Gestational 2-hour plasma glucose (mmol/L) | 6.50 ± 1.45 | 6.57 ± 1.46 | 6.30 ± 1.42 | 0.007 |
| Gestational diabetes |  |  |  | 0.006 |
| No | 856 (82.0%) | 612 (80.0%) | 244 (87.5%) |  |
| Yes | 188 (18.0%) | 153 (20.0%) | 35 (12.5%) |  |
| Maternal hypertension |  |  |  | 0.598 |
| None | 1016 (93.3%) | 747 (93.5%) | 269 (92.8%) |  |
| Pregnancy-induced hypertension | 30 (2.8%) | 21 (2.6%) | 9 (3.1%) |  |
| Pre-eclampsia | 28 (2.6%) | 21 (2.6%) | 7 (2.4%) |  |
| Eclampsia | 2 (0.2%) | 2 (0.3%) | 0 (0.0%) |  |
| Chronic hypertension | 11 (1.0%) | 6 (0.8%) | 5 (1.7%) |  |
| Chronic hypertension superimposed pre-eclampsia | 2 (0.2%) | 2 (0.3%) | 0 (0.0%) |  |
| Parity |  |  |  | 0.221 |
| Parous | 628 (57.3%) | 453 (56.2%) | 175 (60.3%) |  |
| Nulliparous | 468 (42.7%) | 353 (43.8%) | 115 (39.7%) |  |
| Tobacco exposure groups^[[1]](#footnote-2)^ |  |  |  | 0.001 |
| Group 1 | 515 (52.8%) | 395 (56.7%) | 120 (43.0%) |  |
| Group 2 | 284 (29.1%) | 191 (27.4%) | 93 (33.3%) |  |
| Group 3 | 141 (14.4%) | 88 (12.6%) | 53 (19.0%) |  |
| Group 4 | 36 (3.7%) | 23 (3.3%) | 13 (4.7%) |  |
|  |  |  |  |  |
| **Neonatal characteristics** |  |  |  |  |
| Gestational age (wk) | 38.7 ± 1.6 | 38.7 ± 1.7 | 38.9 ± 1.1 | 0.032 |
| Sex |  |  |  | 0.550 |
| Female | 523 (47.8%) | 380 (47.3%) | 143 (49.3%) |  |
| Male | 571 (52.2%) | 424 (52.7%) | 147 (50.7%) |  |
| Birthweight (kg) | 3.08 ± 0.47 | 3.06 ± 0.49 | 3.12 ± 0.38 | 0.054 |
| Fat-free mass (kg) | 2.78 ± 0.31 | 2.87 ± 0.34 | 2.77 ± 0.31 | 0.115 |
| Fat mass (kg) | 0.32 ± 0.14 | 0.35 ± 0.16 | 0.31 ± 0.14 | 0.133 |
| Body fat % | 9.97 ± 3.61 | 10.59 ± 3.88 | 9.91 ± 3.58 | 0.339 |
|  |  |  |  |  |
| **Child Y6 characteristics** |  |  |  |  |
| z-BMI (SDS) | 0.00 ± 1.37 | -0.07 ± 1.35 | 0.17 ± 1.40 | 0.023 |
| z-Height (SDS) | -0.04 ± 0.99 | 0.02 ± 0.98 | -0.17 ± 1.02 | 0.013 |
| Systolic blood pressure (mmHg) | 101 ± 8 | 101 ± 8 | 102 ± 9 | 0.207 |
| Diastolic blood pressure (mmHg) | 60 ± 6 | 60 ± 5 | 60 ± 6 | 0.235 |
| Carotid intima media thickness (mm) | 0.42 ± 0.03 | 0.42 ± 0.03 | 0.42 ± 0.03 | 0.561 |
| Pulse wave velocity(m/s) | 4.95 ± 1.61 | 4.88 ± 1.59 | 5.07 ± 1.62 | 0.206 |

Table S2: Distribution of neonatal abdominal adiposity across the different tertiles of newborn body composition markers (birthweight, fat mass, fat-free mass, body fat %). Values are presented as mean ± standard deviation. Legend: SAT = subcutaneous adipose tissue; IAT = intraabdominal adipose tissue

|  | **SAT (cc)** | **Superficial SAT (cc)** | **Deep SAT (cc)** | **IAT (cc)** |
| --- | --- | --- | --- | --- |
| Birthweight |  |  |  |  |
| Tertile 1 | 71.8 ± 14.7 | 62.0 ± 12.4 | 9.8 ± 3.3 | 18.6 ± 5.7 |
| Tertile 2 | 90.5 ± 20.0 | 77.5 ± 16.5 | 13.0 ± 4.2 | 22.2 ± 6.4 |
| Tertile 3 | 109.7 ± 18.6 | 92.4 ± 15.3 | 17.3 ± 4.9 | 27.0 ± 6.0 |
| Fat-free mass |  |  |  |  |
| Tertile 1 | 75.5 ± 17.8 | 65.0 ± 14.5 | 10.5 ± 4.0 | 18.5 ± 5.7 |
| Tertile 2 | 89.2 ± 20.1 | 76.4 ± 16.5 | 12.8 ± 4.3 | 22.1 ± 5.9 |
| Tertile 3 | 108.9 ± 20.0 | 91.7 ± 16.6 | 17.1 ± 4.9 | 27.4 ± 6.1 |
| Fat mass |  |  |  |  |
| Tertile 1 | 73.2 ± 16.1 | 63.1 ± 13.1 | 10.1 ± 3.6 | 19.5 ± 6.8 |
| Tertile 2 | 90.5 ± 19.3 | 77.3 ± 16.1 | 13.3 ± 4.7 | 23.5 ± 6.3 |
| Tertile 3 | 111.3 ± 17.1 | 94.0 ± 13.8 | 17.3 ± 4.4 | 25.5 ± 6.4 |
| Body fat % |  |  |  |  |
| Tertile 1 | 75.2 ± 17.4 | 64.8 ± 14.5 | 10.4 ± 3.7 | 20.2 ± 7.2 |
| Tertile 2 | 94.3 ± 21.7 | 80.5 ± 18.2 | 13.8 ± 5.0 | 23.4 ± 5.9 |
| Tertile 3 | 108.9 ± 17.9 | 91.8 ± 14.2 | 17.1 ± 4.4 | 25.3 ± 6.6 |

1. Group 1: cotinine <0.17 ng/mL and no environmental tobacco smoke exposure; Group 2: cotinine <0.17 ng/mL but self-reported environmental tobacco smoke exposure; Group 3: cotinine 0.17–13.99 ng/mL (environmental tobacco smoke exposure or light smoking); Group 4: cotinine ≥14 ng/mL (active smoking) [↑](#footnote-ref-2)
